# Planar cell polarity orients airway cilia

**DOI:** 10.1186/2046-2530-1-S1-O20

**Published:** 2012-11-16

**Authors:** E Vladar, JD Axelrod

**Affiliations:** 1 Stanford University School of Medicine, USA

## 

Propulsion of contaminants out of the lungs by coordinated action of motile cilia is essential for airway function. Multiciliated airway epithelial cells undergoing ciliogenesis generate hundreds of basal bodies that traffic to the surface, elongate motile axonemes and align with the tissue axis for coordinated motility. The mechanisms of cilia polarization remain poorly explored. We showed that a conserved set of planar cell polarity (PCP) proteins polarize airway epithelial cilia: PCP proteins localize asymmetrically to the cell cortex, revealing that PCP signaling starts before ciliogenesis; in PCP mutant epithelia, molecular and morphological polarity are disrupted; and PCP mutants display defective airway clearance, highlighting the requirement for ciliary alignment. Studying the cytoskeleton, a target of PCP signaling, we uncovered two temporally-distinct, cell type-specific, planar polarized microtubule (MT) networks. Prior to ciliogenesis, MTs are necessary in all cells for PCP protein asymmetry. Subsequently, after basal bodies dock, MTs are observed to contact the polarized appendages of basal bodies, and PCP-regulated, ciliated cell-specific MTs, stably anchored by their plus ends to the proximal cortex, are required for basal body distribution and alignment. Our evidence suggests that MTs have two separable PCP functions: MTs are first required in every cell for trafficking PCP proteins to the cortex, and later they interact with and directly align cilia. Our studies reveal strong conservation of PCP mechanisms initially described in *Drosophila*, and cilia alignment by PCP-oriented MTs provides the first mechanistic description of how asymmetric PCP proteins at the cortex orient cilia along the cell surface.

